# Silicon Micromachined TSVs for Backside Interconnection of Ultra-Small Pressure Sensors

**DOI:** 10.3390/mi14071448

**Published:** 2023-07-19

**Authors:** Weiwen Feng, Peng Li, Haozhi Zhang, Ke Sun, Wei Li, Jiachou Wang, Heng Yang, Xinxin Li

**Affiliations:** 1 State Key Laboratory of Transducer Technology, Shanghai Institute of Microsystem and Information Technology, Chinese Academy of Sciences, Shanghai 200050, China; fengww@mail.sim.ac.cn (W.F.); 19112020040@fudan.edu.cn (P.L.); haozhi.zhang@mail.sim.ac.cn (H.Z.); sunke@mail.sim.ac.cn (K.S.); weili@mail.sim.ac.cn (W.L.); jiatao-wang@mail.sim.ac.cn (J.W.); h.yang@mail.sim.ac.cn (H.Y.); 2 School of Microelectronics, University of Chinese Academy of Sciences, Beijing 100049, China; 3 State Key Laboratory of ASIC and System, School of Microelectronics, Fudan University, Shanghai 200433, China; 4 Xin-Huangpu Joint Innovation Institute of Chinese Medicine, Guangzhou 510715, China

**Keywords:** pressure sensor, silicon micromachined TSV, backside interconnection, MIS process

## Abstract

This paper presents an ultra-small absolute pressure sensor with a silicon-micromachined TSV backside interconnection for high-performance, high spatial resolution contact pressure sensing, including flexible-substrate applications. By exploiting silicon-micromachined TSVs that are compatibly fabricated with the pressure sensor, the sensing signals are emitted from the chip backside, thereby eliminating the fragile leads on the front-side. Such a design achieves a flat and fully passivated top surface to protect the sensor from mechanical damage, for reliable direct-contact pressure sensing. A single-crystal silicon beam–island structure is designed to reduce the deflection of the pressure-sensing diaphragm and improve output linearity. Using our group-developed microholes interetch and sealing (MIS) micromachining technique, we fabricated ultra-small piezoresistive pressure sensors with the chip size as small as 0.4 mm × 0.6 mm, in which the polysilicon-micromachined TSVs transfer the signal interconnection from the front-side to the backside of the wafer, and the sensor chips can be densely packaged on the flexible substrate via the TSVs. The ultra-small pressure sensor has high sensitivity of 0.84 mV/kPa under 3.3 V of supply voltage and low nonlinearity of ±0.09% full scale (FS) in the measurement range of 120 kPa. The proposed pressure sensors with backside-interconnection TSVs hold promise for tactile sensing applications, including flexible sensing of wearable wristwatches.

## 1. Introduction

Micromechanical piezoresistive pressure sensors have played important roles in various fields, such as smart devices, automotive electronics, industrial applications and aerospace [1,2,3]. As a type of pressure sensor, the absolute pressure sensor can directly measure pressure instead of differential pressure. Therefore, this type of sensor has an irreplaceable role in some application scenarios [4,5,6,7,8]. Among these roles, high spatial resolution pressure sensing has become a critical technology for many applications, such as tactile sensing and medical pulse detection [9,10]. These applications place very high demands on the sensor chip size and packaging method of the sensors.

Traditional piezoresistive pressure sensors are fabricated based on (100) bulk silicon micromachining techniques, employing the backside etching technique to create the pressure reference chamber and pressure-sensitive diaphragm [11,12]. The pressure reference chamber is sealed and evacuated with the wafer bonding process. However, anisotropic wet etching of silicon inevitably results in outwardly expanded sidewalls in the pressure reference cavity, thereby causing difficulties in reducing the sensor chip size. Alternatively, backside deep reactive ion etching (DRIE) can be used for fabricating the pressure-sensing structure, offering the advantage of chip-size reduction [13,14,15]. However, the non-uniformity of the etching rate of DRIE affects device consistency, and the high processing cost limits mass production and application. Surface-micromachined piezoresistive pressure sensors can be fabricated through thin-film deposition and sacrificial layer removing techniques, thereby reducing both the size and cost by eliminating the backside etching and bonding process [16]. However, their sensitivity is much lower due to the utilization of polysilicon piezoresistors, which exhibit a piezoresistive coefficient approximately 50% lower than that of single-crystal silicon. In addition, the residual stress in the thin-film diaphragm degrades the sensing performance. By leveraging silicon–on–insulator (SOI) wafers and utilizing the buried oxide layer as a sacrificial layer, compact pressure sensors with single-crystal silicon piezoresistors can be formed with a single-sided process [17]. The high cost of SOI wafers and the complicated process to define the diaphragm edge impose limitations on the widespread adoption of these sensors. Therefore, the development of high-performance and ultra-small size single-crystal silicon piezoresistive pressure sensors holds great significance.

Another important issue with pressure sensors lies in packaging techniques. Many recently developed wearable applications need to use tiny-sized pressure sensors or dense sensor arrays, such as senseless blood pressure measurement bracelets and traditional Chinese medicine pulse diagnosis wristwatches, in which the very small sensor chips need to be packaged on flexible substrates. The conventional front-side wire-bonding process easily causes broken wires or electric leakage onto the skin. To mitigate the impact of liquids and measured pressure on wire bonding, a thick layer of silicone gel is applied to the device surface, aiming to isolate the wires from the measured object. However, this solution compromises the sensitivity and consistency of sensors within the array [10]. Furthermore, the wire bonding technique requires additional space on a flexible printed circuit board (FPCB) for placing solder pads, resulting in a reduction in sensor array density. Therefore, the interconnection technology of front-side wire bonding limits the application of silicon-based pressure sensors in high spatial resolution pressure sensing domains. Apparently, through-silicon via (TSV) interconnection technology [18] is a good solution for flexible applications of pressure sensors, with which chip-sized packaging can be achieved, and the interconnection is performed on the backside of the sensor chip. However, normal copper electroplating is used for the TSV technique, which is frequently used in 3D integrated circuit (IC) chips [19,20] or sensor–IC heterogeneous integration microsystems [21,22]. Such TSV processes are not very compatible with the silicon-micromachined tiny-size pressure sensor chips, the polishing and flattening of which can easily damage the sensing diaphragm structure. TSVs are expected to be formed still using the silicon-micromachining process, which is compatible with the fabrication of the pressure sensor chips.

In this work, we propose an ultra-small pressure sensor (chip size = 0.4 mm × 0.6 mm) with a silicon-micromachined TSV backside interconnection. Employing TSVs, the sensor signal goes out from the backside, realizing a flat and fully passivated top surface. This design eliminates the influence of external force on the package and improves the performance of contact pressure detection. Using our group-developed microholes interetch and sealing (MIS) micromachining process in a (111) silicon wafer, we achieved the fabrication of ultra-small pressure sensors with silicon-micromachined TSVs for packaging on the flexible substrate.

## 2. Sensor Design and Modeling

**Figure 1:**
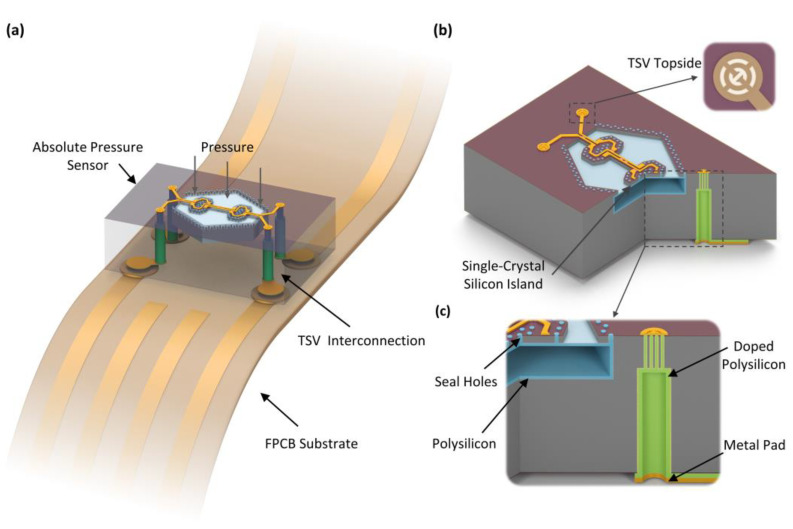
Schematic diagrams of the ultra-small pressure sensor with silicon-micromachining TSV package of backside interconnection. (**a**) Schematic of the sensor chip packaged on a flexible substrate. (**b**) Partial cross-section view of the sensor chip. (**c**) Close-up view of the silicon-micromachining process formed TSV.

The ultra-small pressure sensor chip with a TSV backside interconnection package to a flexible PCB substrate is schematically illustrated in Figure 1a. The silicon sensor chip body is transparent to reveal the internal structure. The sensor consists of a pressure-sensing diaphragm, a vacuum chamber for absolute pressure reference and four TSVs for backside interconnection. The pressure-sensing diaphragm comprises a single-crystal silicon beam-island structure on top of a flat polysilicon thin-film diaphragm. Four single-crystal silicon piezoresistors are distributed within the single-crystal silicon beams. The pressure reference chamber is evacuated and vacuum sealed to provide a pressure reference close to a vacuum. The power supply and the output signal of the sensor are transmitted through the four TSVs to the backside of the sensor chip, where they can be connected to an FPCB via four solder pads.

A partial cross-section view of the three-dimensional sensor structure is shown in Figure 1b. A 2.5 μm-thick polysilicon layer is inside the entire pressure reference chamber, with its upper thin film suspended as the pressure-sensitive diaphragm. To minimize the nonlinearity induced by excessive deflection of the polysilicon membrane under pressure, an 8 μm-thick single-crystal silicon beam-island structure is designed on its top. Three beams are designed in the center and on the double sides, where the pressure-induced stress is concentrated. A piezoresistive Wheatstone bridge for pressure detection is integrated into the single-crystalline silicon beams. The two islands are designed in the areas between the beams to enhance the stress concentration at the beams. The release and sealing of the suspended sensing structure are formed by anisotropic etching through dozens of 5 μm-diameter microholes specifically designed in the structure. These microholes are used for wet etching and sealing. The pressure reference chamber is wet etched through the microholes. Polysilicon is then deposited to seal the microholes, simultaneously sealing the pressure reference chamber. To reduce the negative influence of the sealed microholes on the mechanical response of the sensor to pressure, the holes are all distributed surrounding the edge of the diaphragm and in the single-crystal silicon islands. The sensor is fabricated using the MIS process. Taking advantage of the crystal orientation distribution of (111) silicon wafers, the process utilizes a combination of anisotropic wet etch and sidewall vertical dry etch to create the single-crystal silicon structure with the suspended thin-film and the vacuum chamber beneath. The chamber and the diaphragm created by the MIS process in (111) silicon have a hexagonal shape. The technical details of the (111) silicon micromachining method can be found in [23,24], in which our group developed microholes interetch and sealing (MIS) process, and its updated version of thin-film under bulk-silicon (TUB) was addressed.

As illustrated in Figure 1b,c, the TSV structure consists of two segments from the double sides of the chip. The upper portion consists of several concentric annular high-aspect-ratio grooves with a depth of 30 μm, and the lower part is a deep and wide hole with a diameter of 36 μm. The two parts are connected through the silicon substrate thickness of 420 μm to guide out the signal lines from the front-side to the backside of the sensor. To achieve an electrical connection, n-type doped polysilicon is deposited onto the sidewalls of the TSV, and isolation is achieved by an oxide layer between the polysilicon and the bulk silicon. On the front-side of the sensor, polysilicon seals the annular deep grooves, and a metal line layer is formed on top of the polysilicon to guide out the piezoresistive Wheatstone bridge. At the bottom of the sensor, the polysilicon extends to the backside and forms the solder pads for TSV packaging.

To reduce the TSV resistance, the doped polysilicon conductive layer is designed to be 2 μm thick. However, in the upper part of the TSV, the thickness of the conductive layer is determined by the width of the annular grooves. Therefore, the annular grooves are designed as wide as 4 μm to achieve a balance between conductance and groove refilling. The area of the TSV upper structure is 224 μm^2^. According to the measured resistivity of the doped polysilicon of about 9 mΩ·cm, the calculated resistance of a single TSV can be about 120 Ω. Thanks to the much higher input impedance of normal amplifiers for the signal interface, such a level of TSV resistance is well acceptable.

**Figure 2:**
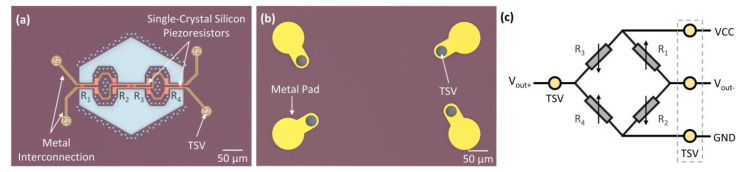
Schematics of the designed piezoresistors and metal pads. (**a**) Front-side design of the sensor. (**b**) Backside design. (**c**) Diagram of piezoresistive Wheatstone bride.

Figure 2a,b illustrates the front-side and backside of the sensor chip. Four piezoresistors are arranged on the single-crystal silicon beam, with resistors R_1_ and R_4_ located at the double sides and resistors R_2_ and R_3_ located in the middle beam, corresponding to the pressure-induced tensile stress region and the compressive stress region, respectively. A single piezoresistor is designed with a width of 2 μm and a length of 52 μm, presenting a serpentine arrangement. The (111) single-crystal silicon piezoresistive sensitivity can be calculated by performing a coordinate transformation of piezoresistive coefficients [25]. The longitudinal and transverse piezoresistive coefficients are calculated as π_44_/2 and -π_44_/6, respectively. The four piezoresistors are designed to form a fully sensitive Wheatstone bridge, as shown in Figure 2c. When subjected to pressure, R_1_ and R_4_ exhibit opposite resistance variations, while R_2_ and R_3_, are negative, resulting in differential output. For pressure measurement, we set R_1_ = R_4_ and R_2_ = R_3_. Upon an applied excitation voltage V_in_, the piezoresistors will change, and ΔR_1_ = ΔR_4_, ΔR_2_ = ΔR_3_. According to the principle of the Wheatstone bridge, the resistance change of the piezoresistor will be transformed into the voltage of the output port. The output voltage of the bridge can be expressed as:

(1) $$V_{\mathrm{out}}=V_{\mathrm{out}+}-V_{\mathrm{out}-}=\frac{1}{2}\left|\frac{\Delta R_{1}}{R_{1}}-\frac{\Delta R_{2}}{R_{2}}\right|V_{\mathrm{in}}$$

**Figure 3:**
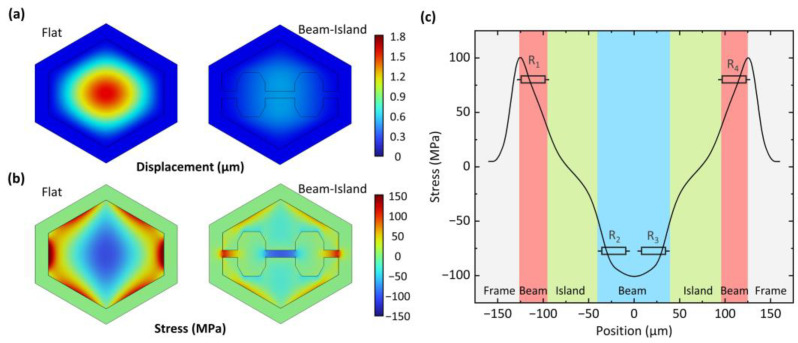
Finite-element simulation of the designed beam–island–diaphragm pressure sensing structure compared to an identical-sized conventional flat-membrane pressure sensing structure. (**a**) Displacement of the two structures under 100-kPa pressure. (**b**) Stress distributions. (**c**) For the beam–island–diaphragm structure, simulated stress distribution along the beam direction is plotted for piezoresistor arrangement.

To verify the design of the beam–island–diaphragm sensing structure and obtain the stress distribution to optimize the piezoresistive arrangement, a finite-element simulation model for a polysilicon flat-membrane structure is compared to this single-crystal silicon beam–island reinforced polysilicon diaphragm structure. The model includes a 1:1 scale pressure-sensitive structure and a silicon frame. To reduce the complexity of the simulation, structures such as substrates, leads and pressure reference chambers that have nearly no influence on the mechanical properties of pressure-sensitive structures are removed. When 100 kPa of pressure is applied, the displacements of the two structures are compared in Figure 3a. The flat-membrane structure exhibits a maximum deflection of about 1.8 μm, which is quite large and comparable to the diaphragm thickness of 2.5 μm. Such large deflection will cause significant sensing nonlinearity [25]. In contrast, the maximum deflection of the single-crystal silicon beam–island reinforced structure is only about 0.4 μm, which is much smaller than the thickness of the diaphragm and can effectively eliminate output nonlinearity. The stress distributions of the two pressure-sensitive structures are compared in Figure 3b. The beam–island–diaphragm structure can concentrate stress within the beam region, thereby achieving the maximum stress, which is similar to that of the flat-membrane structure. Therefore, the beam–island–diaphragm design can effectively reduce nonlinearity while maintaining high sensitivity for pressure detection. The stress distribution along the beam direction is simulated and shown in Figure 3c. Based on the stress distribution, the piezoresistors are designed to be the same length as the beam, maximizing their utilization of stress.

## 3. Micromachining Fabrication

**Figure 4:**
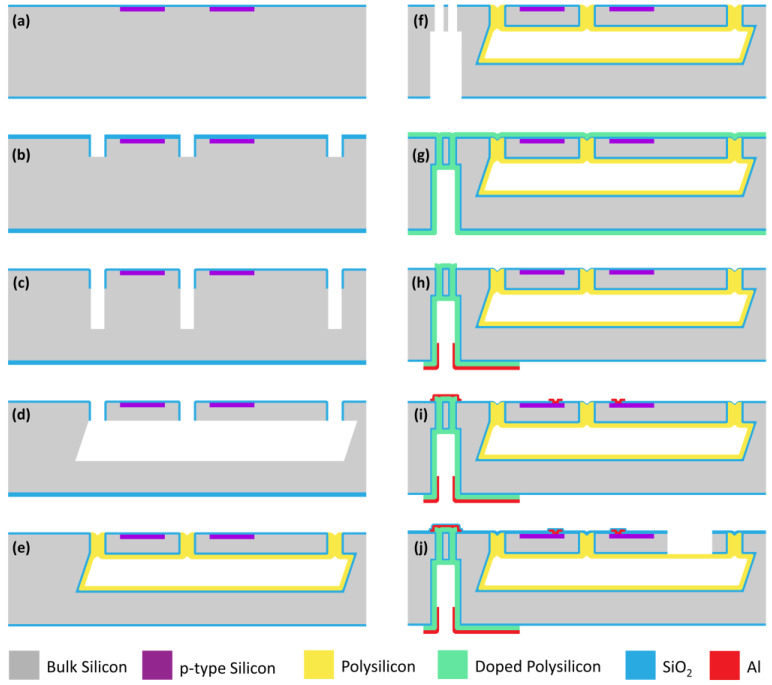
Fabrication process of the TSV backside–interconnected pressure sensor. (**a**) Piezoresistance ion implantation and dielectric film deposition. (**b**) Beam–island structure patterning. (**c**) Deep RIE etching to open the microholes. (**d**) TMAH wet etching. (**e**) Polysilicon deposition and surface removal. (**f**) Front-side etching for TSVs. (**g**) Doped polysilicon deposition. (**h**) Metal pad patterning on the backside. (**i**) Metal patterning on the front side. (**j**) Passivation layer deposition and etching to form beam islands.

Based on our developed MIS micromachining technology, the designed ultra-small pressure sensors with a silicon-micromachined TSV backside interconnection are fabricated. The MIS technique enables the processing of the tiny-sized sensor fabrication together with TSV backside interconnection. Moreover, this approach utilizes general (111) wafers instead of SOI wafers, resulting in a cost reduction. The fabrication steps of the sensor start with a double-side polished 4-inch (111) wafer. The detailed steps are illustrated in Figure 4 and are described as follows.

(a) Thermal oxidation is performed on a (111) silicon wafer to generate a 150 nm-thick SiO_2_ layer. Boron ions are area-selectively implanted to create p-type piezoresistors. The implantation energy is 40 keV, and the dose is 3.5 × 10^15^ ion/cm^2^. Then, the impurities are activated by annealing. According to the simulation results of the doping process, the range of ion implantation is about 0.4 μm, indicating the thickness of the piezoresistors.
(b) The microhole trenches are patterned by photolithography, and the SiO_2_ is etched to expose the bulk silicon by reactive ion etching (RIE). Subsequently, the silicon is etched to 8 μm in depth to determine the thickness of the single-crystal silicon beam-islands with deep RIE (DRIE). Then, 400 nm-thick SiO_2_ is deposited by low-pressure chemical vapor deposition (LPCVD) and is anisotropically etched by RIE to cover the trench surface. Then, the SiO_2_ at the trench bottom is removed by RIE for subsequent DRIE. The SiO_2_ sidewall is used to protect the single-crystal silicon structures against the subsequent wet etch.
(c) Trenches that are 20 μm deep are further etched using DRIE to define the depth of the pressure reference chamber.
(d) Tetramethylammonium hydroxide (TMAH) at a 25% concentration is used to perform the anisotropic wet etch at 80 °C. Due to the etching principles to (111) silicon, the lateral etch finally forms the pressure reference chamber. The etching process creates a hexagonal chamber enclosed by eight (111) crystal-plane sidewalls. The etching can be automatically stopped when the (111) sidewalls are exposed.
(e) Thermal oxidation is performed to generate a 120 nm-thick SiO_2_ layer to cover all the surface, including the inside of the chamber. Then, 2.5 μm-thick polysilicon is deposited using LPCVD to form a 2.5 μm-thick film inside the chamber as a pressure-sensitive diaphragm. Simultaneously, the trenches of the microholes are sealed into a near vacuum. During the polysilicon deposition, the pressure inside the LPCVD furnace is controlled at 30 Pa. After the process, the wafers are cooled to room temperature, and the pressure inside the sealed pressure reference chamber will be less than 10 Pa, which is an approximate vacuum. Subsequently, the polysilicon on the surface is removed by RIE to eliminate the surface protrusions.
(f) Double-sided patterning and etching of TSVs are next. The fabrication of the TSVs is carried out in two separate steps. First, the trenches on the front side of the wafer are patterned and etched using DRIE. The etching depth is set to 30 μm. Then, the vias on the backside are patterned and etched by DRIE until the front-side trenches are exposed.
(g) Thermal oxidation is performed to generate a 150 nm-thick SiO_2_ layer for electrical insulation of the TSVs. Then, phosphorus-doped polysilicon with a thickness of 1.5 μm is deposited by LPCVD [26,27]. The PH_3_ as-deposited doped polysilicon covers the inner wall of the TSV and refills the trenches on the front-side, thus realizing the electrical connection between the two sides. The resistivity of the doped polysilicon is measured at about 9 mΩ·cm.
(h) The polysilicon on the top surface is removed by RIE, while the polysilicon in the TSV regions is retained to enhance the contact with the metal. Aluminum at 1 μm-thick is sputtered on the backside of the wafer. The aluminum pads are patterned, and the polysilicon pattern is formed using ion beam etching and RIE.
(i) The contact holes of the single-crystal silicon piezoresistors are patterned by removing SiO_2_ with RIE. The interconnections between the piezoresistors and the TSVs are formed by sputtering and patterning an aluminum layer.
(j) SiO_2_ at 300 nm thick is deposited on the front side using plasma-enhanced CVD as the passivation layer. Finally, the beam–island structure is formed by masked etching of SiO_2_/silicon/SiO_2_ with RIE, thereby realizing the structural release of the pressure-sensitive structure.

**Figure 5:**
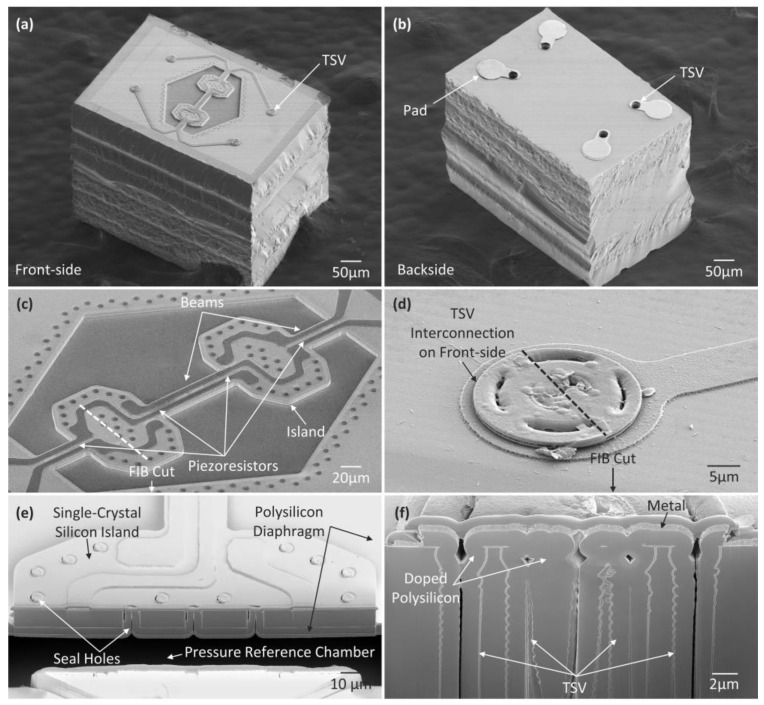
SEM images of the ultra-small pressure sensor chip with silicon-micromachined TSV backside interconnection: (**a**,**b**) Front side and backside of the sensor chip. All pads are fabricated on the backside and connected by TSVs. (**c**) Details of pressure-sensing diaphragm; (**d**) Details of the TSV on the front side; (**e**) Cross-sectional view of the beam–island structure; (**f**) Cross-sectional view of the upper portion of the silicon-micromachined TSV.

The fabricated pressure sensors with silicon-micromachined TSV backside interconnection are characterized using scanning electron microscopy (SEM) and focused ion beam (FIB) microscopy. The size of the whole sensor chip is 0.4 mm × 0.6 mm. As illustrated in Figure 5a,b, all the pads are transformed from the front side to the chip backside for interconnecting the pressure sensor chips on flexible substrates with the TSVs. Figure 5c,e shows the magnified and cross-sectional views of the pressure-sensing diaphragm, in which the single-crystal silicon beam–island structure is fabricated on the flat polysilicon diaphragm. Figure 5d provides a close-up view of the TSV on the front-side of the sensor. Figure 5f provides a cross-sectional view of the upper portion of the silicon micromachined TSV. The grooves of the TSV are filled with doped polysilicon, providing electrical interconnection.

**Figure 6:**
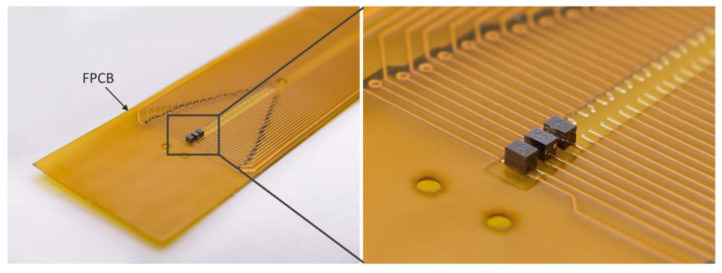
Optical images of the packaged ultra-small pressure sensors with silicon-micromachined TSV backside interconnection.

Figure 6 shows the packaged pressure sensors with silicon micromachined TSV interconnection. The sensor pads on the backside are directly bonded and electrically connected to the solder pads of FPCB using conductive silver paste on the backside. The exposed and protruding leads are eliminated by the TSV backside interconnection in the packaged sensors, improving reliability for the pressure measurements.

## 4. Test Results

We mounted the packaged sensor in an airtight chamber to perform pressure tests. The airtight chamber was connected to an air pressure-generating system formed by a hand-controlled pump (Druck PV211, Druck Inc., Leicester, UK) and a standard pressure gauge (Druck DPI 104, with an accuracy of ±0.05% FS). The airtight chamber, together with the sensor, was placed in a thermostatically controlled test chamber to attain a controllable testing environment with an adjusted temperature. During the pressure test, the sensor was operated under a DC voltage of 3.3 V. The output voltage of the sensor was read out using a multimeter (Keysight 34470A, Santa Rosa, CA, USA), and the output voltage was not amplified.

**Figure 7:**
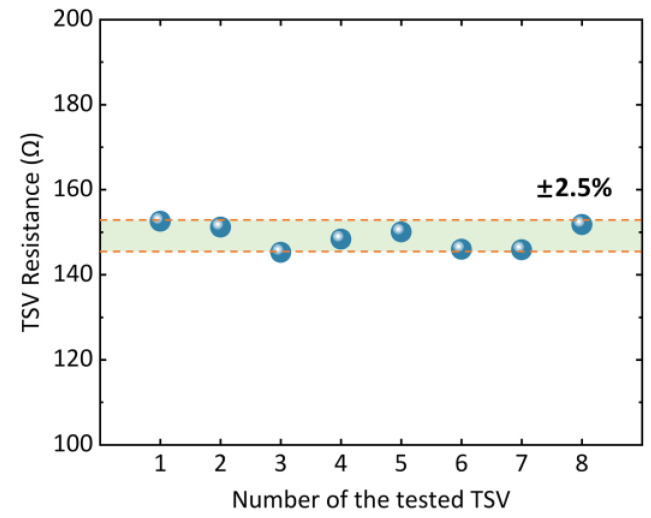
Testing results of the TSV resistance show that the resistance of the silicon-micromachined TSV is about 150 Ω, with the deviation within ±2.5%.

As shown in Figure 7, the interconnection properties of the silicon-micromachined TSVs are measured. The resistance of a single TSV is about 150 Ω, which generally agrees with the design. The small difference from the designed value comes from the difference in resistivity of doped polysilicon and the change in pattern during TSV deep hole processing. The resistance deviation among randomly selected TSVs in one wafer is within ±2.5%. The TSVs are connected in series to the signal path, causing a slight offset in the operating voltage of the sensor. However, as a common-mode signal, such offset voltage can be eliminated in the output signal from the Wheatstone bridge. Moreover, the output resistance of the piezoresistive Wheatstone bridge (including TSVs) is measured as about 9.9 kΩ. The resistance of the TSV is far smaller than the piezoresistive Wheatstone bridge; thus, the influence of the resistance of the TSVs can be ignored.

**Figure 8:**
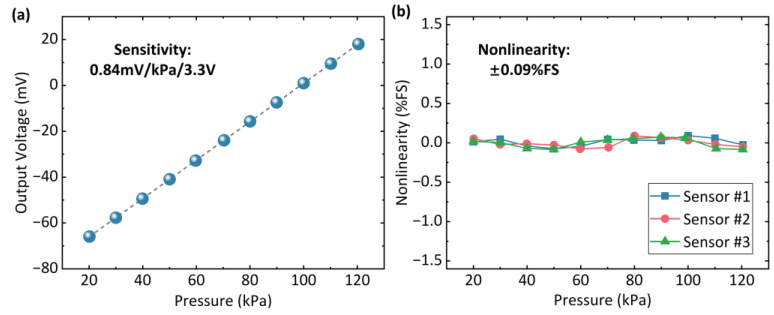
Output response of the pressure sensor with TSV backside interconnection. (**a**) The sensitivity of the pressure sensor. (**b**) The nonlinearity of the pressure sensor.

The pressure-sensing performance is tested by changing the pressure inputs from 20 kPa to 120 kPa at 25 °C, with the results shown in Figure 8a. The sensitivity of the sensor is 0.84 mV/kPa under 3.3 V of supplied voltage. According to the resistance of the sensor, the noise equivalent pressure of the sensor can be calculated as 0.014 Pa. Based on the pressure-testing results, the nonlinearity of the sensor can be calculated as ±0.09% full scale (FS), demonstrating good linearity of the output response, as illustrated in Figure 8b.

**Figure 9:**
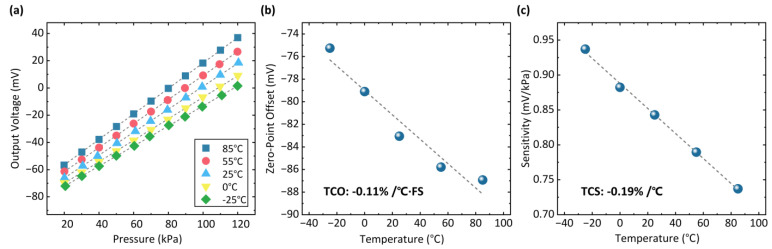
Testing results of the pressure sensor under pressure inputs at different temperatures ranging from −25 °C to 85 °C. (**a**) Pressure-sensing responses of the sensor at different temperatures. (**b**) The temperature coefficient of offset of the sensor. (**c**) The temperature coefficient of sensitivity of the sensor.

To test the temperature characteristics of the sensor, the pressure sensing performance is repeatedly tested at different temperatures, as shown in Figure 9a. The zero offset and sensitivity of the sensor at different temperatures are calculated, as shown in Figure 9b,c. Within the temperature range of −25 °C to 85 °C, the temperature coefficient of offset (TCO) is −0.11%/°C·FS, and the sensitivity temperature coefficient of sensitivity (TCS) is −0.19%/°C, indicating normal temperature stability of the ultra-small piezoresistive pressure sensor with the silicon-micromachined TSVs for vertical interconnection.

## 5. Conclusions

In summary, we designed an ultra-small pressure sensor with a silicon-micromachined TSV backside interconnection. Utilizing silicon-micromachined TSVs, the sensor signal goes out from the backside, achieving a flat and fully passivated top surface. This design eliminates the exposed fragile leads, thus improving the reliability and performance of the sensor in contact pressure measurements. Based on the MIS process, we achieved the fabrication of ultra-small pressure sensors (0.4 mm × 0.6 mm). In the pressure range of 120 kPa, the sensor has a high sensitivity of 0.84 mV/kPa under 3.3 V of supply voltage and low nonlinearity of ±0.09% FS. Meanwhile, the sensor has a TCO of −0.11%/°C·FS and a TCS of −0.19%/°C over a temperature range of −25 °C to 85 °C. The ultra-small pressure sensor with silicon-micromachined TSV backside interconnection holds promise for a wide range of applications in high-performance, high spatial resolution, contact-based pressure-sensing fields.

## Data Availability

Not applicable.
